# The Effect of Race and Chronic Obstructive Pulmonary Disease on Long-Term Survival after Coronary Artery Bypass Grafting

**DOI:** 10.3389/fpubh.2013.00004

**Published:** 2013-04-15

**Authors:** Jimmy T. Efird, Wesley T. O’Neal, Curtis A. Anderson, Jason B. O’Neal, Linda C. Kindell, T. Bruce Ferguson, W. Randolph Chitwood, Alan P. Kypson

**Affiliations:** ^1^Department of Cardiovascular Sciences, East Carolina Heart Institute, Brody School of Medicine, East Carolina UniversityGreenville, NC, USA; ^2^Center for Health Disparities Research, Brody School of Medicine, East Carolina UniversityGreenville, NC, USA; ^3^Department of Internal Medicine, Wake Forest University School of MedicineWinston-Salem, NC, USA; ^4^Department of Anesthesia, Critical Care and Pain Medicine, Beth Israel Deaconess Medical Center, Harvard Medical SchoolBoston, MA, USA

**Keywords:** COPD, CABG, survival, long-term, race

## Abstract

**Background:** Chronic obstructive pulmonary disease (COPD) is a known predictor of decreased long-term survival after coronary artery bypass grafting (CABG). Differences in survival by race have not been examined.

**Methods:** A retrospective cohort study was conducted of CABG patients between 2002 and 2011. Long-term survival was compared in patients with and without COPD and stratified by race. Hazard ratios (HR) and 95% confidence intervals (CI) were computed using a Cox regression model.

**Results:** A total of 984 (20%) patients had COPD (black *n* = 182; white *n* = 802) at the time of CABG (*N* = 4,801). The median follow-up for study participants was 4.4 years. COPD was observed to be a statistically significant predictor of decreased survival independent of race following CABG (no COPD: HR = 1.0; white COPD: adjusted HR = 1.9, 95% CI = 1.7–2.3; black COPD: adjusted HR = 1.6, 95% CI = 1.1–2.2).

**Conclusion:** Contrary to the expected increased risk of mortality among black COPD patients in the general population, a similar survival disadvantage was not observed in our CABG population.

## Introduction

Chronic obstructive pulmonary disease (COPD) affects an estimated 15 million Americans and is an important predictor of mortality following coronary artery bypass grafting (CABG) (1, 2, 3, 4). The prevalence of COPD among CABG patients varies from 11 to 25.8% (1, 2, 4). COPD recently has become the third leading cause of death in the United States (5). Risk factors potentially associated with cardiovascular disease and COPD include history of smoking, increasing age, exposure to air pollution, and lower socioeconomic position, with smoking being the lead risk factor (6, 7). Between 1980 and 2000, COPD death rates increased by 67% in whites and 87% in blacks even though COPD is more prevalent in whites (6).

Survival paradoxes are well-documented in the cardiovascular literature. Conventional cardiovascular risk factors such as black race, hypercholesterolemia, hypertension, and obesity are associated with increased survival among some patient populations (8, 9). For example, a recent examination of the national Society of Thoracic Surgeons (STS) Adult Cardiac Surgery Database reported a survival advantage among obese patients after CABG compared with non-obese patients (9).

The rationale for the current study was to determine if similar reverse epidemiologic findings are observed among black COPD patients undergoing isolated CABG in our rural, racially dichotomous population. Consistent with the expected force of mortality among blacks, we hypothesized that white COPD patients would have better long-term survival than black COPD patients (6).

## Materials and Methods

### Study design

This was a retrospective cohort study of patients undergoing first-time, isolated CABG at the East Carolina Heart Institute between 2002 and 2011. Demographic data, comorbid conditions, coronary artery disease (CAD) severity, and surgical data were collected at the time of surgery. Patients with COPD were compared with those without COPD. Only black and white patients were included to minimize the potential for residual confounding (∼1% other races). Racial identity was self-reported. Emergent cases were considered a clinically different population with a different etiology following surgery and were excluded in our analysis (*n* = 105). The study was approved by the Institutional Review Board at the Brody School of Medicine, East Carolina University.

### Definitions

COPD was classified based on severity using the following criteria at the time of surgery: Mild: FEV1 60–75% of predicted, and/or on chronic inhaled or oral bronchodilator therapy; Moderate: FEV1 50–59% of predicted, and/or on chronic steroid therapy aimed at lung disease; Severe: FEV1 < 50% predicted, and/or room air pO_2_ < 60 or room air pCO_2_ > 50. Mortality was defined as any cause of death postoperatively. CAD was defined as at least 50% stenosis and confirmed by angiography before surgery.

### Setting

The East Carolina Heart Institute is a 120-bed cardiovascular hospital located in the center of eastern North Carolina, a rural region with a large black population. Cardiovascular disease is the number one cause of death in North Carolina with an unequal burden occurring in eastern North Carolina (10). The institute is a population-based tertiary referral center. Nearly all patients treated at the East Carolina Heart Institute live and remain within a 150 mile radius of the medical center.

### Data collection and follow-up

The primary sources of data extraction were the STS Adult Cardiac Surgery Database and the electronic medical record at the Brody School of Medicine.

Cardiovascular surgery information at our facility has been reported to the STS since 1989. Data quality and cross-field validation are routinely performed by the Epidemiology and Outcomes Research Unit at the East Carolina Heart Institute. An electronic medical record was introduced at the Brody School of Medicine in 1997. Local and regional clinics were consolidated under a single electronic medical record in 2005 which allowed for efficient patient follow-up. The electronic medical record system applies multiple logic comparisons to reliably reduce mismatching of patient data across clinics and follow-up visits. The STS database is linked to the electronic medical record through a unique patient medical record number. COPD status was not collected in our database prior to 2002.

The National Death Index was used to obtain death dates for patients lost to follow-up and also used to validate death information captured in our electronic medical record (11, 12, 13). Linkage with the National Death Index was based on a multiple criteria, deterministic matching algorithm (13). In our database, less than 5% of validated deaths failed to correctly match with the National Death Index.

### Statistical analysis

Categorical variables were reported as frequency and percentage while continuous variables were reported as mean ± standard deviation, median, and range. Variables not previously categorized were divided into quartiles prior to statistical analysis. Quartile categorization is advantageous because it limits the influence of outliers and allows for the assessment of trend across categories. Follow-up time was measured from the date of surgery to the date of death or censoring. Survival probabilities were computed using the Kaplan–Meier product limit method and stratified by COPD and race. The log-rank test was used to compare survival between patients with and without COPD and among COPD patients by race. Cox proportional hazard regression models were used to compute hazard ratios (HR) and 95% confidence intervals (CI) for long-term mortality. The initial multivariable models included variables that have been previously reported to be associated with cardiovascular-related mortality, regardless of their statistical significance in our dataset. These included age, sex, race, hypertension, CAD severity, congestive heart failure (CHF), and prior stroke. The *post hoc* addition of other variables into the model was performed in a pairwise fashion. The test statistic of Grambsch and Therneau was used to check the proportional hazards assumption (14). Statistical significance for categorical variables was tested using the chi-square (χ^2^) method and the Kruskal–Wallis procedure for continuous variables. *P*_Trend_ was computed using a likelihood ratio test. Temporality during the study period was assessed by stratifying the analysis by two time periods.

Few values were missing (<1% for included variables). However, when values were missing they were entered into the regression models as a separate category. A sensitivity analysis with missing values excluded also was performed to confirm that model beta coefficients did not substantively differ from the above results.

Statistical significance was defined as *p* < 0.05. SAS Version 9.3 (Cary, NC, USA) was used for all analyses.

## Results

A total of 984 (20%) patients had COPD (black *n* = 182; white *n* = 802) at the time of CABG (*N* = 4,801). The prevalence of COPD was higher in white vs. black patients (21 vs. 18%) (*p* < 0.05). The severity of COPD among black vs. white patients, defined as none, mild, moderate/severe, was not statistically different (*p* = 0.10). Patient characteristics are described in Table 1. The median follow-up for study participants was 4.4 years.

**Table 1:** **Patients characteristics and univariable survival after CABG (*N* = 4,801)**.

Characteristic	No COPD *n* (%)	COPD		Univariable HR (95% CI)
		Black *n* (%)	White *n* (%)	
Overall	3,817 (80)	182 (4)	802 (16)	2.1 (1.8–2.4)^§^
Age (years)				
Q1 (≤56)	987 (26)	53 (29)	178 (22)	1.0 Referent
Q2 (>56–64)	995 (26)	43 (24)	223 (28)	1.5 (1.1–1.9)
Q3 (>64–72)	998 (26)	46 (25)	218 (27)	2.3 (1.9–2.9)
Q4 (>72)	837 (22)	40 (22)	183 (23)	3.9 (3.2–4.9)
Mean ± SD, median (range)	64 ± 10, 64 (26–90)	63 ± 10, 63 (33–85)	64 ± 10, 65 (31–89)	*P*_Trend_ < 0.0001
Sex				
Male	2,723 (71)	117 (64)	595 (74)	1.0 Referent
Female	1,094 (29)	65 (36)	207 (26)^†^	1.3 (1.1–1.5)
Race				
White	2,967 (78)	–	–	1.0 Referent
Black	850 (22)	–	–	1.2 (1.03–1.4)
BMI (kg/m^2^)*				
Obese (≥30)	1,716 (45)	78 (43)	314 (39)	1.0 Referent
Overweight (25–29.9)	1,461 (38)	64 (35)	268 (33)	1.3 (1.1–1.5)
Normal (18.5–24.9)	622 (16)	34 (19)	204 (25)	1.8 (1.5–2.2)
Underweight (<18.5)	18 (<1%)	6 (3)	16 (2)^††^	2.5 (1.4–4.4)
Mean ± SD, median (range)	30 ± 5.7, 29 (13–66)	29 ± 6.3, 29 (17–53)	29 ± 6.0, 28 (15–51)^††^	*P*_Trend_ < 0.0001
Status				
Elective	1,908 (50)	73 (40)	351 (44)	1.0 Referent
Non-elective	1,908 (50)	109 (60)	451 (56)^††^	1.2 (1.04–1.4)
CAD severity				
1 Vessel	268 (7)	10 (5)	41 (5)	1.0 Referent
2 Vessel	971 (25)	41 (23)	218 (27)	1.6 (1.1–2.4)
3 Vessel	2,578 (68)	131 (72)	543 (68)	1.9 (1.4–2.8)
				*P*_Trend_ < 0.0001
Left main disease				
No	2,870 (75)	127 (70)	555 (69)	1.0 Referent
Yes	947 (25)	55 (30)	247 (31)^††^	1.1 (0.93–1.3)
Recent smoker				
No	2834 (74)	85 (47)	362 (45)	1.0 Referent
Yes	983 (26)	97 (53)	440 (55)^††^	0.98 (0.85–1.1)
Hypertension				
No	680 (18)	16 (9)	122 (15)	1.0 Referent
Yes	3,137 (82)	166 (91)	680 (85)^††^	1.4 (1.2–1.7)
Diabetes				
No	2,309 (60)	94 (52)	490 (61)	1.0 Referent
Yes	1,508 (40)	88 (48)	312 (39)	1.4 (1.3–1.7)
Congestive heart failure				
No	2,927 (77)	107 (59)	532 (66)	1.0 Referent
Yes	890 (23)	75 (41)	270 (34)^††^	2.1 (1.9–2.5)
Renal failure				
No	3,744 (98)	175 (96)	771 (96)	1.0 Referent
Yes	73 (2)	7 (4)	31 (4)^††^	4.2 (3.2–5.6)
Dialysis				
No	3,730 (98)	165 (91)	793 (99)	1.0 Referent
Yes	87 (2)	17 (9)	9 (1)^††^	4.9 (3.7–6.4)
Peripheral arterial disease				
No	3,320 (87)	136 (75)	590 (74)	1.0 Referent
Yes	497 (13)	46 (25)	212 (26)^††^	2.1 (1.8–2.5)
Prior MI				
No	2,007 (53)	77 (42)	367 (46)	1.0 Referent
Yes	1,810 (47)	105 (58)	435 (54)^††^	1.5 (1.3–1.7)
Prior stroke				
No	3,509 (92)	157 (86)	719 (90)	1.0 Referent
Yes	308 (8)	25 (14)	83 (10)^††^	1.8 (1.5–2.3)
Prior PCI				
No	2,876 (75)	135 (74)	619 (77)	1.0 Referent
Yes	941 (25)	47 (26)	183 (23)	0.83 (0.70–0.98)

^†^*p* < 0.05; ^††^*p* < 0.01; χ^2^ (Categorical variables), Kruskal–Wallis Test (Continuous variables). *Missing category not shown. ^§^COPD vs. no COPD. BMI, body mass index; CABG, coronary artery bypass grafting; CAD, coronary artery disease; CI, confidence interval; COPD, chronic obstructive pulmonary disease; HR, hazard ratio; MI, myocardial infarction; PCI, percutaneous coronary intervention; Q1, quartile 1; Q2, quartile 2; Q3, quartile 3; Q4, quartile 4.

COPD patients had a lower body mass index (BMI) (mean = 29 ± 6.0 vs. 30 ± 5.5; *p* < 0.01) and were more likely to be white (82 vs. 78%; *p* < 0.05) than non-COPD patients. On admission, COPD patients were more likely to have left main disease (31 vs. 25%), hypertension (86 vs. 82%), CHF (35 vs. 23%), renal failure (4 vs. 2%), peripheral arterial disease (PAD) (26 vs. 13%), previous myocardial infarction (MI) (55 vs. 47%), and previous stroke (11 vs. 8%) (*p* < 0.01). COPD patients also were more likely to be recent smokers (55 vs. 26%) and have non-elective CABG (57 vs. 50%) (*p* < 0.01).

**Figure 1:**
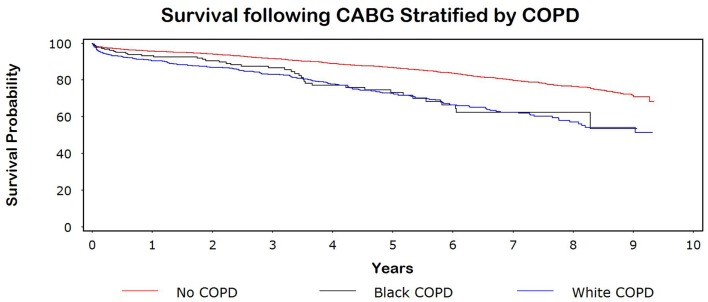
**CABG, coronary artery bypass grafting; COPD, chronic obstructive pulmonary disease**.

Kaplan–Meier unadjusted survival curves are shown in Figure 1. The 5-year survival for patients with and without COPD was 73 and 87%, respectively (*p* < 0.0001). The 5-year survival for black and white COPD patients was similar (73%).

The unadjusted HR for COPD was 2.1 (95% CI = 1.8–2.4) compared with no COPD. After adjusting for age, sex, race, hypertension, CAD severity, CHF, and prior stroke, the HR decreased to 1.9 (95% CI = 1.6–2.2). COPD was observed to be a statistically significant predictor of decreased survival independent of race following CABG (no COPD: HR = 1.0; white COPD: adjusted HR = 1.9, 95% CI = 1.7–2.3; black COPD: adjusted HR = 1.6, 95% CI = 1.1–2.2) (Table 2). The multivariable results did not substantively change with the pairwise addition of other variables listed in Table 1.

**Table 2:** **Multivariable survival after CABG**.

Characteristic	Multivariable HR (95% CI)
Main effect
No COPD	1.0 Referent
Black COPD	1.6 (1.1–2.2)
White COPD	1.9 (1.7–2.3)
Age (years)
Q1 (≤56)	1.0 Referent
Q2 (>56–64)	1.4 (1.1–1.8)
Q3 (>64–72)	2.2 (1.7–2.7)
Q4 (>72)	3.5 (2.8–4.3)
	*P*_Trend_ < 0.0001
Sex
Male	1.0 Referent
Female	1.1 (0.92–1.2)
CAD severity
1 Vessel	1.0 Referent
2 Vessel	1.4 (0.97–2.1)
3 Vessel	1.5 (1.1–2.2)
	*P*_Trend_ = 0.017
Hypertension
No	1.0 Referent
Yes	1.2 (1.02–1.5)
Congestive heart failure
No	1.0 Referent
Yes	1.8 (1.6–2.1)
Prior stroke
No	1.0 Referent
Yes	1.5 (1.3–1.9)

*CABG, coronary artery bypass grafting; CAD, coronary artery disease; CI, confidence interval; COPD, chronic obstructive pulmonary disease; HR, hazard ratio*.

## Discussion

The significantly increased risk of mortality among COPD patients after CABG in our study (adjusted HR = 1.9, 95% CI = 1.6–2.2) is consistent with previous reports (1, 2, 4). A review of 33,137 CABG cases from hospitals in northern New England found COPD to be an independent predictor of decreased long-term survival (HR = 1.8, 95% CI = 1.6–2.1) (1). A covariate-matched analysis of 3,760 patients in New York that underwent isolated CABG similarly found an increased risk in long-term mortality for patients with COPD (HR = 1.28, 95% CI = 1.11–1.47) (2). Furthermore, a review of 13,337 CABG procedures in the United Kingdom found COPD to be a predictor of long-term survival and differences were noted between moderate (HR = 1.3, 95% CI = 1.1–1.5) and severe (HR = 1.9, 95% CI = 1.5–2.4) cases (4). Our results also are comparable with population-based data showing that COPD is a predictor of mortality in the general population and that COPD is more prevalent in white patients (6, 15).

Death rates have been reported to be higher among black vs. white COPD patients, reflecting a greater force of mortality among blacks in the general population (6, 16). A recent examination of the national lung transplant list observed that black COPD patients died sooner than whites; however, the results were confounded by patients who were removed from the transplant list (17). In contrast, the HR for the black COPD arm in our study was in the reverse direction, being less than white COPD. In a comparable analysis of patients without COPD, an increased force of mortality was observed among black PAD patients (no PAD, HR = 1.0; white PAD: adjusted HR = 1.4, 95% CI = 1.1–1.8; black PAD: adjusted HR = 2.1, 95% CI = 1.5–3.0). The latter highlights a clear force of mortality among black patients for another chronic disease population at-risk for cardiovascular disease and emphasizes the unexpected finding among black COPD patients in our study.

Survival paradoxes in black patients are well-documented in the literature (18, 19, 20, 21). Our data showed no survival advantage for white COPD patients and a survival paradox for black COPD patients cannot be ruled out. Contrary to our hypothesis, a statistically significant survival disadvantage was not observed among black patients in our CABG population. However, uncertainty remains due to the limited number of patients at-risk toward the end of the study. A possible explanation for our discordant findings may involve differences in lung function and susceptibility to tobacco smoke between black and white COPD patients (22, 23). Also, differences in the response to standard COPD therapies could exist between races (24).

### Strengths and limitations

Our study is strengthened by its comparatively large sample size and long-term follow-up. To the best of our knowledge, this is the largest study to date examining survival differences among COPD CABG patients by race. Furthermore, we were able to accurately determine time of death using a combination of the National Death Index and our comprehensive electronic medical record.

Another strength of this study is its target base. A large priority population in eastern North Carolina allowed for us to report on a group that has experienced historic differences in socioeconomic position and discrimination. Twenty-eight (97%) of the 29 counties in eastern North Carolina fall below the national per capita income of $27,915, with half reporting a value less than $20,000 (25). Similarly, 90% of the counties have a higher percentage of blacks than the national value of 13.1% (25). Our results are generalizable to other low-income, rural, and racially diverse populations.

Pulmonary function tests were not repeated prior to surgery. Accordingly, there may have been misclassification of COPD status between patients. We were unable to stratify our analyses by COPD severity and race because of a limited number of patients. Survival after CABG among COPD patients has been shown to vary by disease severity and racial differences could exist across the COPD severity spectrum (4).

Socioeconomic position, education, and income were not collected and these factors may have influenced survival (26). Payor status, which has been shown in some studies to predict survival independent of race, was not consistently collected and consequently was not used in our analysis (27). Additionally, we were unable to reliably estimate socioeconomic position using zip codes because a large percentage of patients in our catchment area live in rural areas with postal box addresses.

Patients in this study were recruited over a relatively long period (10 years), over which practice methods and clinical care may have changed considerably. However, results were consistent throughout the study after stratifying by two time periods, indicating the robustness of the data to temporal changes. The status of several variables in our analysis may have changed over time. We did not adjust for these variables in a time-dependent manner due to their potential to be in the causal pathway. Similarly, surgical complications and medication use were not included in our analysis because of their time-dependent status.

Cause of death is not recorded in the National Death Index and COPD status may have been unrelated to their mortality. Although we adjusted for known clinically relevant variables, we acknowledge that other unmeasured factors could have influenced our results due to the retrospective nature of this study. Retrospective studies also are susceptible to recall and selection bias. We cannot rule out that the association between COPD and poor survival is non-causal in nature and may be due to non-cardiac causes unassociated with comorbidities or surgical procedures such as exacerbations requiring hospitalizations (15). Additionally, examining the right tail of the Kaplan–Meier curve (Figure 1), it is observed that the number of individuals at-risk reduces to a few patients after year 6. Probability estimates at these later time points may not be reliable and must be interpreted with caution.

We considered missing values to be a distinct category and they were entered into the regression models as a separate category rather than being imputed. Imputation methods require data to be “missing at random” which is difficult to verify given the sparseness and unknown distribution of the missing values (28, 29). We cannot rule out misclassification bias due to grouping missing values into a distinct category although such bias likely is trivial given the small number of missing values. Furthermore, we performed a complete case analysis with missing values removed and this did not substantively alter our results. We opted not to report the complete analysis because this would have reduced power for our multivariable analysis. Removing values only from the multivariate analysis also would have resulted in a different dataset being analyzed.

Our use of quartile boundaries, while desirable for minimizing the influence of outliers, may have yielded overly broad categories and the potential for residual confounding. However, the substitution of continuous variables in our models did not materially alter results. Except for race, we did not examine interactions among other clinically relevant variables included in our dataset. Given the large number of potential multi-level interactions involving the independent variables in our analyses, it is difficult to interpret such effects. Furthermore, we did not use regression-based tests for interaction because they are known to have weak power and often fail to detect interactions when they exist (30). Multivariable Cox regression models, rather than propensity score matching, were used to control for confounding because of potential “non-collapsibility bias” inherent to logistic regression-based propensity scores and the possible loss of power due to incomplete matching (31). Alternative methods such as machine learning (e.g., random forest algorithm) may introduce misspecification into the propensity score model due to the “black box” nature of the algorithm that obscures the etiologic relationship between predictors and outcome and were not used in the current analysis (32, 33).

## Conclusion

Contrary to the expected increased risk of mortality among black COPD patients in the general population, a similar survival disadvantage was not observed in the current study. Future research is needed to confirm our initial findings. Exploring the association between preoperative conditions such as COPD and outcomes, especially in the context of racial disparities in rural populations, may help guide future decisions regarding appropriate clinical follow-up and surgical referral.

## Conflict of Interest Statement

The authors declare that the research was conducted in the absence of any commercial or financial relationships that could be construed as a potential conflict of interest.
